# The Brief Case: *Legionella micdadei* caught red-handed

**DOI:** 10.1128/jcm.00302-25

**Published:** 2025-08-13

**Authors:** Priyanka Gupta, Hiranmayi Vemaganti, Nahed Ismail, Vijayalakshmi (Viju) Ananthanarayanan

**Affiliations:** 1 Department of Pathology, University of Illinois at Chicago 14681, Chicago, Illinois, USA; 2 Department of Pathology, Clinical Microbiology, Laboratory Medicine, University of Illinois at Chicago, University of Illinois Health System, Chicago, Illinois, USA; 3 Department of Pathology, University of Illinois Hospital and Clinics/University of Illinois at Chicago 14681, Chicago, Illinois, USA; Mayo Clinic Minnesota, Rochester, Minnesota, USA

**Keywords:** *Legionella micdadei*, acid-fast bacilli (AFB), immunocompromised hosts, Bronchoalveolar lavage (BAL)

## CASE

A 64-year-old female presented to the emergency department with 3 days of fever with intermittent chills, dry cough, nausea, and vomiting. She was recently diagnosed with mantle cell lymphoma and started on chemotherapy with bendamustine-rituximab. The patient had completed the sixth cycle of her chemotherapy 3 days prior to the current emergency room visit.

On admission, she was febrile (40.30°C) and mildly dehydrated without pronounced respiratory symptoms. Initial laboratory workup revealed leukopenia (2.9 k/µL), absolute lymphopenia (0.5 k/µL), and low electrolytes (sodium 129 mmol/L and potassium 3.2 mmol/L). The chest X-ray was significant for multiple new focal nodular lesions distributed in both lungs. A previous positron emission tomography scan done 3 months prior to current admission was negative for metabolically active lung lesions. However, a chest CT scan on admission showed multiple bilateral pulmonary soft tissue masses, measuring up to 4.1 cm in greatest dimension in the left upper lobe, raising suspicion for an infectious or a neoplastic process.

Due to a high clinical index of suspicion for invasive fungal and bacterial infections, empiric antifungal and antibiotic therapy of isavuconazonium sulfate and ceftriaxone was initiated.

Bronchoalveolar lavage (BAL) was performed. BAL fluid (550 cells/µL) consisted primarily of neutrophils, alveolar macrophages, and other mononuclear inflammatory cells, with no significant evidence of viral cytopathic changes or malignant epithelial cells. Significant hemosiderin-like pigment was observed in less than 5% of the macrophages. The following laboratory tests were negative: Cytomegalovirus culture from BAL fluid; urine antigens for *Histoplasma capsulatum*, *Blastomyces* species, and *Legionella pneumophila* serogroup 1 (1, 3); β-D-glucan, Aspergillus galactomannan from serum and BAL, TB QuantiFERON Gold, and HIV (fifth generation antigen-antibody) in peripheral blood; and respiratory virus panel testing of nasopharyngeal swabs by PCR. Aerobic, anaerobic, fungal, mycobacterial, other viral cultures, and direct fluorescent antibody (DFA) test for *Pneumocystis jirovecii* in BAL were negative. Testing of sputum revealed normal respiratory flora without any findings on stains (Gram and acid-fast bacilli [AFB]) and cultures.

**Fig 1:**
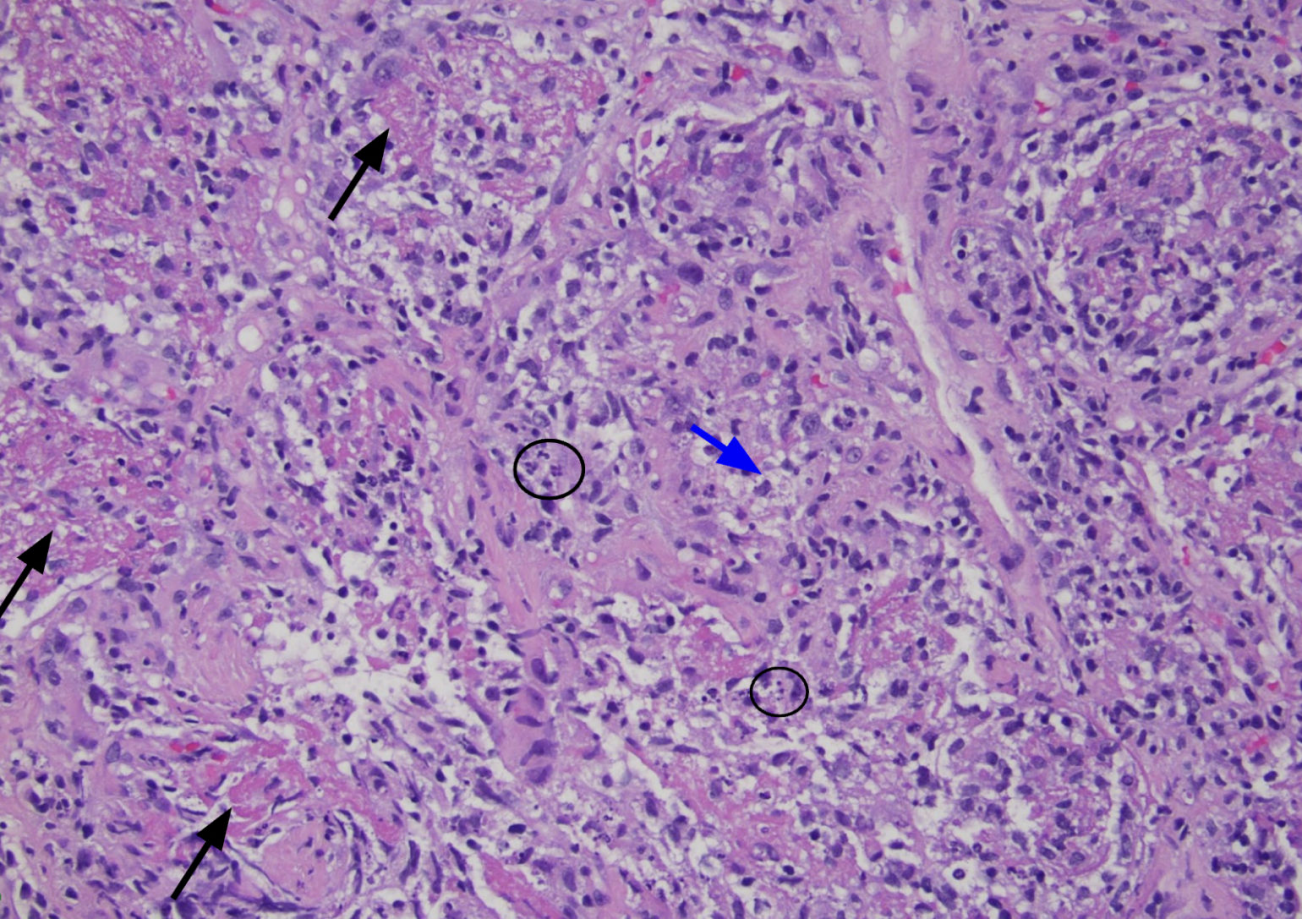
Intra-alveolar fibrinous exudates (black arrows), histiocytes (blue arrow), and karyorrhectic debris (circled), consistent with features of acute fibrinous and organizing pneumonia.

**Fig 2:**
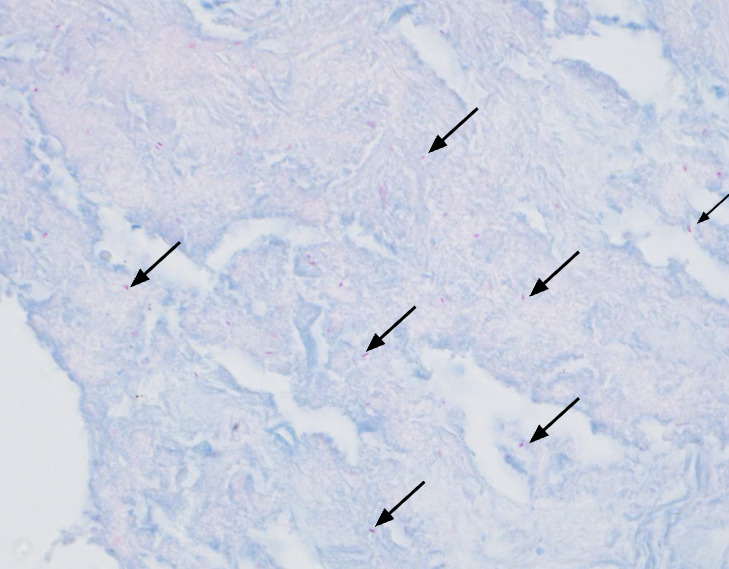
Single scattered AFB (under oil immersion).

Further work-up with transbronchial biopsy of the pulmonary nodules was performed. Hematoxylin and eosin-stained sections showed inflammation of lung parenchyma with intra-alveolar fibrinous exudates with histiocytes and karyorrhectic debris consistent with changes of acute fibrinous and organizing pneumonia (Fig. 1). Additionally, Kinyoun AFB staining of lung biopsy tissue revealed many acid-fast bacilli scattered singly and in small clusters (Fig. 2). Gram stain and culture (aerobic, AFB, and buffered charcoal yeast extract [BCYE]) were not performed on the biopsy specimen due to a lack of request from the clinical team. Formalin-fixed, paraffin-embedded tissue was sent to Washington University for 16sRNA PCR analysis, and organisms were identified as *Legionella micdadei*. The empirical antimicrobial treatment continued, and the patient was discharged in a stable state.

## DISCUSSION

*L. micdadei* is the second most common *Legionella* species after *L. pneumophila*, affecting humans and accounting for 9% of all *Legionella* infections in the United States (1, 2). It was first identified in 1979 as an opportunistic pathogen causing pneumonia in an immunocompromised group of patients in Pittsburgh and was named the “Pittsburgh pneumonia agent” (3). Originally proposed as *Legionella pittsburghensis*, the organism was subsequently reclassified as *L. micdadei* (4). It typically infects lower respiratory airways and is an etiological agent for both community-acquired and hospital-acquired pneumonia and lung abscesses (2). It is also implicated as a causative agent in numerous non-pulmonary infections like brain abscesses, prosthetic valve endocarditis, prosthetic joint infections, and necrotizing cellulitis, possibly due to its propensity for a hematogenous spread after an initial respiratory infection (5–8). Similar to other *Legionella* species, *L. micdadei* tends to colonize water supplies like shower heads and piping systems and is known to cause nosocomial infections in immunocompromised patients through infected water supplies (9). At present, in the USA, the Centers for disease control mandates nationwide reporting so that appropriate infection control measures like source identification and decontamination are implemented in a timely fashion to curtail its transmission, especially in the vulnerable immunocompromised population (10).

*L. micdadei* is an aerobic, fastidious, facultative intracellular, gram-negative bacterium. They are difficult to visualize on Gram stain, which can be attributed to their intracellular location within macrophages and the presence of necrotic background tissue—features commonly observed in pulmonary infections caused by *Legionella*. In our case, the Gram stain in BAL was negative. Furthermore, these organisms can exhibit acid-fast staining on primary smears and can initially be mistaken for mycobacterial organisms. Interestingly, their acid-fast property is lost upon culture in an enriching medium (2). It is important to note that *Legionella* is longer (approximately 0.3–0.9 µm wide and 2–20 µm long) when compared to *Mycobacterium tuberculosis* (0.3–0.9 µm wide and 1.5–4 µm long) (11, 12). The absence of growth in AFB cultures further indicated that these organisms were not *Mycobacterium*, emphasizing the need to consider *L. micdadei* in cases of acid-fast organisms in respiratory specimens.

Laboratory diagnosis of *L. micdadei* in clinical laboratories can be missed due to several reasons, including the selective growth requirements. *L. micdadei* shows preferential growth in standard BCYE medium containing L-cysteine, iron, and alpha-ketoglutarate (13). Enrichment of BCYE with bovine albumin is thought to enhance the growth of *L. micdadei* and some strains of *L. bozemanae*, but enrichment practices are not used universally (14). Isolation of *Legionella* from contaminated sources such as environmental specimens or sputum is usually attempted by supplementation of BCYE with antibiotic combinations containing polymyxin-A, anisomycin, vancomycin, or cycloheximide. It is noteworthy that antibiotics can further inhibit *Legionella* growth and can lead to lower recovery rates than desired. Culture plates are typically incubated at 35°C–37°C in an atmosphere of 2.5% carbon dioxide in the air for 7–10 days. Initial growth from the specimen can be observed in 3–5 days or longer, but after a few passes, it grows well within 48 hours (13). Unfortunately, the tissue sample from our patient was not sent for microbiological analysis (Gram stain, culture, and AFB stain/culture). The AFB stain was done on paraffin-embedded tissue sections as a part of the workup following histological examination of the tissue that demonstrated fibrinous organizing pneumonia-like features. Other conventional commercial methods, such as rapid urine antigen tests, available for the detection of *L. pneumophila* are not deemed suitable for the detection of *L. micdadei*. As observed in our patient, standard urine antigen tests that are designed to detect the soluble antigen of *L. pneumophila* serogroup 1 do not detect other *L. pneumophila* serogroups or other *Legionella* species (15). Antibody-based assays such as DFA and slide agglutination tests (SATs) were considered critical for the detection of *L. pneumophila* during earlier outbreaks due to rapid identification but were unable to discriminate between *L. pneumophila* and non-pneumophila spp. While the DFA assay can be performed on serum, tissues, and secretions, SATs require a pure culture isolate. Although DFA is a sensitive and specific assay for the diagnosis of Legionnaires’ disease, these methods are not currently employed in clinical laboratories for routine identification as they are fraught with several practical challenges, including the lack of readily available reagents, technical difficulty, and potential cross-reactivity of antibodies against *Legionella* species. Additionally, DFA testing, as a serological method, is suboptimal for the early detection of acute *Legionella* infection due to several inherent limitations. First, defining a diagnostic threshold for a single elevated antibody titer is problematic, as 5%–10% of the general population exhibits high baseline titers. Second, accurate diagnosis often depends on demonstrating seroconversion, typically a fourfold rise in IgG antibody levels between acute and convalescent serum samples collected 4–8 weeks apart, making the approach unsuitable for timely clinical decision-making. Despite these constraints, serological assays have been used in the past and may retain utility in epidemiological studies and in scenarios where conventional diagnostic tools such as culture, urinary antigen testing, or molecular methods fail to identify the pathogen (16).

To date, the most reliable and definite methods of detection of *Legionella* species are molecular amplification and detection and include 16S ribosomal RNA (full or partial gene) sequencing, *mip* or *rpoB* gene sequencing, and whole-genome sequencing employed for the identification of *L. micdadei*. Recently, Matrix assisted laser desorption/ionization- Time of flight (MALDI-TOF) has also shown encouraging results in the successful identification of *Legionella* species. A recent study suggested that MALDI Biotyper can help to identify the non-*L*. *pneumophila* species by processing the colonies from L-cysteine-enriched BCYE media, followed by the direct extraction method (17).

In the present case, the inability to culture *Legionella* in a clinical laboratory can be explained by multiple factors: a low index of suspicion by the clinician, which did not trigger the appropriate microbiological testing protocol; inadequate sampling, as BAL fluid was collected from the right lung as opposed to the organisms being recovered from the left lung on tissue biopsy; and prophylactic antibiotic exposure of the patient at the time of testing.

The Kinyoun stain performed on transbronchial biopsy demonstrated acid-fast organisms on tissue sections but failed to do so in sputum and BAL. A higher identification of *L. micdadei* in tissue biopsy is possible owing to its intracellular localization within tissue macrophages, which also explains a low recovery rate in upper respiratory specimen*s*. A competent immune system is capable of eliminating the infection via infected macrophage-mediated, inflammasome-dependent IL-1 release that activates bystander immune cells. This drives the production of critical cytokines and facilitates infection control (18). In a weakened immune system, as in hematological malignancies, AIDS, or diabetes mellitus, a defective cellular immunity predisposes to ineffective elimination of the organism and thereby progression of disease. Solid organ transplantation, tumor necrosis factor-alpha inhibitors, and anti-CD52 agents have also been reported to increase the risk of clinical infection (19, 20).

*L. micdadei* exhibits susceptibility to quinolones and newer macrolides, which require low dosing frequency and have excellent bioavailability and hence make them the antimicrobials of choice. While both trimethoprim-sulfamethoxazole (TMP-SMX) and cefepime can be used in the treatment of *Legionella* infections, these are typically not first-line therapy choices (20). TMP-SMX is a commonly used antibiotic combination for prophylaxis against *Pneumocystis jirovecii* pneumonia and other opportunistic infections in immunocompromised patients, particularly those with HIV, or receiving immunosuppressive therapy (e.g., after solid organ transplant or for hematological malignancies), or on high-dose steroids. Interestingly, our patient, before admission, was taking TMP-SMX thrice a week as part of a prophylactic regimen and continued without modification until the day of discharge (20).

*L. micdadei* is a known nosocomial agent, but in the present case, the patient did not have any prior hospital admissions or medical procedures that would raise suspicion for nosocomial infection. She developed a respiratory illness prior to her current hospital admission. Since the temporal association argued against a possible nosocomial infection, an infection control protocol for source identification and decontamination was not activated at this time. Methods like thermal control and chemical disinfection are some of the commonly used and effective strategies to eliminate the bacterial burden in the water system. Other infection control measures, such as isolation and airborne precautions, are not applicable for *Legionella* infections, as the disease is not known to spread between humans and can be controlled through environmental monitoring of water sources (9).

In conclusion, *L. micdadei* infections should be considered in the differential diagnosis of pulmonary infections with positive AFB staining, especially in immunocompromised hosts. A high index of clinical suspicion and consequent employment of appropriate methods of detection can increase microbial recovery, leading to the timely implementation of optimal antimicrobial therapy and effective infection control measures.

## SELF-ASSESSMENT QUESTIONS

1. All are true regarding *L. micdadei* infections except:
  - Nosocomial spread through water supplies
  - Can cause pulmonary as well as non-pulmonary infections
  - Urine antigen test for L. *pneumophila* serotype 1 can be used for its detection
  - 16S rRNA PCR is a highly accurate diagnostic test
2. The following culture medium is used for the isolation of *L. micdadei*:
  - Buffered charcoal yeast agar (BCYE)
  - Thiosulfate citrate bile salts sucrose (TCBS) agar
  - Lowenstein Jensen (LJ) medium
  - Thayer Martin agar
3. What infection control measures are recommended for hospital-acquired respiratory infections caused by *L. micdadei*?
  - Airborne
  - Contact
  - Droplet
  - Environmental monitoring

## ANSWERS TO SELF-ASSESSMENT QUESTIONS

1. All are true regarding *L. micdadei* infections except:
  - Nosocomial spread through water supplies
  - Can cause pulmonary as well as non-pulmonary infections
  - Urine antigen test for *L. pneumophila* serotype 1 can be used for its detection
  - 16S rRNA PCR is a highly accurate diagnostic test

Answer: c. Urine antigen test for *L. pneumophila* serotype 1 can be used for its detection.

Methods of detection for *L. legionella* like urine antigen test cannot be used for *L. micdadei* infections. Molecular methods like 16S rRNA amplicon sequencing are definitive methods of diagnosis.

- The following culture medium is used for the isolation of *L. micdadei*:
  - Buffered charcoal yeast agar (BCYE)
  - Thiosulfate citrate bile salts sucrose (TCBS) agar
  - Lowenstein Jensen (LJ) medium
  - Thayer Martin agar

Answer: a. Buffered charcoal yeast agar (BCYE).

Buffered charcoal yeast agar containing L-cysteine, iron, and alpha-ketoglutarate, which are growth requirements for *L. micdadei*. TCBS agar, LJ medium, and Thayer Martin medium are used for isolation of *Vibrio* spp., *Mycobacterium*, and *Neisseria gonorrhoeae*, respectively.

- What infection control measures are appropriate for hospital-acquired respiratory infections with *L. micdadei*?
  - Airborne
  - Contact
  - Droplet
  - Environmental monitoring

Answer: d. Environmental monitoring.

Other infection control measures such as isolation and airborne precautions applicable to other respiratory infections are not applicable for infections due to Legionella. It is not known to spread between humans and can be controlled with environmental monitoring of water sources.

**TAKE-HOME POINTS**

- *L. micdadei* is an emerging pathogen capable of both nosocomial and community transmission, primarily affecting individuals with compromised cell-mediated immunity.
- Diagnosis can be challenging as it exhibits poor Gram staining and weak acid-fast properties.
- Legionella infections are nationally notifiable and must be reported to state and local health authorities.
- Prevention focuses on environmental monitoring and decontamination of potential sources, such as cooling towers and showerheads.
